# Hybrid Repair of Infected Femoral Artery Pseudoaneurysm: Stent Graft Placement and Artificial Graft Replacement

**DOI:** 10.7759/cureus.65657

**Published:** 2024-07-29

**Authors:** Hirotaka Yamauchi, Soichiro Kageyama, Akinori Kojima, Hideo Morita, Takeki Ohashi

**Affiliations:** 1 Cardiovascular Surgery, Nagoya Tokushukai General Hospital, Kasugai, JPN

**Keywords:** minimally invasive procedure, artificial blood vessel replacement, endovascular stent graft, hybrid procedures, infected femoral artery pseudoaneurysm

## Abstract

A femoral artery pseudoaneurysm is the most prevalent complication of femoral access due to the artery's accessibility and frequent use for catheterization and blood tests. An infected femoral artery pseudoaneurysm is often life-threatening and challenging to manage. A 70-year-old male with a history of tongue cancer treatments, including resection, lymph node dissection, and radiation chemotherapy, visited his previous physician for a fever and was prescribed oral antibiotics, but the fever persisted, accompanied by pain and a mass in the left groin. An enhanced CT revealed an infected pseudoaneurysm of the left femoral artery. The fever's etiology was unclear but likely stemmed from a blood draw from the femoral artery during a prior visit, resulting in a pseudoaneurysm that became infected. The patient was transferred to our hospital due to management challenges. Blood cultures from the previous hospital were positive, and laboratory tests indicated an active infection. The initial strategy was to continue antibiotic therapy to control the infection. After approximately a month of antibiotic treatment, blood cultures remained negative, and laboratory results improved significantly. However, the aneurysm had clearly enlarged, necessitating emergency surgery. Typically, surgical intervention requires opening the abdomen to replace the external iliac artery to its extent, a considerably invasive procedure for the patient. Thus, we opted for a hybrid treatment, implanting a stent graft from the external iliac artery to the proximal common femoral artery and replacing artificial blood vessels from there to the femoral artery bifurcation. The postoperative course was favorable. In this case, we provided the optimal treatment for the patient's condition, despite the impossibility of a radical cure due to the cancer's progression. We believe the infected pseudoaneurysm was adequately controlled, and the hybrid therapy is effective for patients who cannot endure more invasive treatments.

## Introduction

The femoral artery is highly accessible and frequently utilized for catheterization and blood tests. A pseudoaneurysm of the femoral artery is the most prevalent complication at the femoral access site, arising post-catheterization, anastomosis of the native artery and artificial graft, trauma, or infection [1]. Notably, infected femoral artery pseudoaneurysm (IFAP) is often life-threatening and challenging to manage. Treating IFAP necessitates open surgical repair, which carries a high complication rate [2-4].

Recent advancements have made endovascular stent-graft placement an effective and minimally invasive alternative technique for aneurysm treatment, particularly as a temporary bridge for critically ill patients [5].

In this report, we describe a case of IFAP caused by a blood draw puncture to the groin, despite the infection source being unknown. A hybrid repair was performed to minimize invasiveness for a patient with carcinoma in situ who was at high risk. This treatment may be beneficial for patients who are unsuitable for highly invasive procedures.

## Case presentation

A 70-year-old male patient was transferred to our department for further evaluation and management of an infected pseudoaneurysm of the left femoral artery, occurring during chemotherapy for tongue cancer. The patient had previously undergone tongue resection and lymph node dissection for tongue cancer, resulting in a tracheotomy and gastrostomy. Additionally, a central venous port was implanted for chemotherapy and radiation therapy. However, due to persistent cancer recurrences, the patient was reluctant to continue the current chemotherapy regimen.

**Figure 1:**
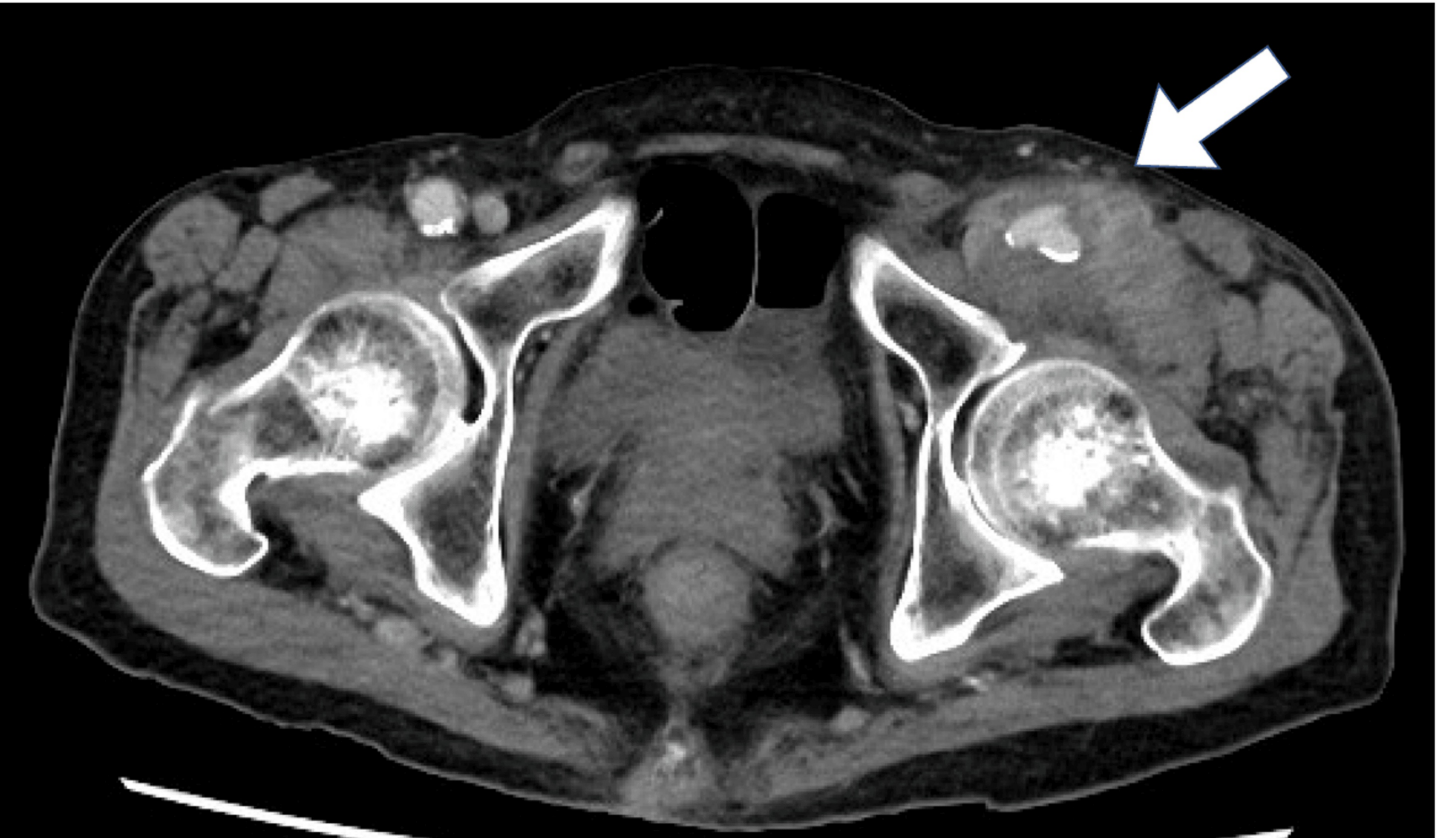
The previous hospital's enhanced CT shows a pseudoaneurysm with diameter of 41 x 50 mm The arrow indicates the failed vessel wall and the leaking blood, which is compressing the surrounding tissue. CT: computed tomography

During a visit to his previous physician for a fever, he was prescribed amoxicillin, an oral antibiotic. Despite this, the fever persisted, and pain accompanied by a mass in the left groin was observed. A CT scan indicated the presence of inguinal cellulitis, and the antibiotic regimen was adjusted to levofloxacin. Three days later, the patient presented with a pulsatile mass and worsening pain. Enhanced CT imaging confirmed the diagnosis of an infected pseudoaneurysm of the left femoral artery (Figure 1).

The Duke classification was not met except for blood culture, including UCG, infectious endocarditis was negative, and the etiology of the fever was unclear. It was hypothesized that a blood draw from the femoral artery during the previous visit led to the formation of a pseudoaneurysm that subsequently became infected. Consequently, surgical intervention was deemed necessary, and the patient was referred to our hospital 10 days after the initial visit to the previous physician. Methicillin-sensitive *Staphylococcus aureus* was identified in the blood culture at the previous hospital, and antibiotic treatment was switched to intravenous cefazolin.

Upon arrival, the patient was alert with a blood pressure of 108/69 mmHg, a heart rate of 71 beats per minute, a respiratory rate of 16 breaths per minute, and a body temperature of 36.6°C. Laboratory tests showed elevated C-reactive protein, indicating an active infection (Table 1).

**Table 1:** Laboratory analysis results on admission to our hospital HDL: high-density lipoprotein, LDL: low-density lipoprotein, HbA1c: hemoglobin A1C, NGSP: National Glycohemoglobin Standardization Program

Laboratory analysis	Results	Normal range
White blood cell counts (/μL)	7,600	3,300-8,600
Hemoglobin (g/dL)	11.9	13.7-16.8
Platelet count (×10^3^/μL)	473	158-348
Creatine kinase (U/L)	26	59-248
Lactate dehydrogenase (U/L)	171	124-222
Aspartate aminotransferase (U/L)	80	13-30
Alanine aminotransferase (U/L)	39	10-42
γ-glutamyl transpeptidase	45	13-64
Total bilirubin (mg/dL)	0.2	0.4-1.5
Direct bilirubin (mg/dL)	0.1	0.1-0.3
Alkaline phosphatase (U/L)	126	38-113
Amylase (mg/dL)	61	44-132
Creatinine (mg/dL)	0.58	0.65-1.07
Blood urea nitrogen (mg/dL)	13.7	8.0-20.0
Uric acid (mg/dL)	2.3	3.7-7.8
Sodium (mEq/L)	139	138-145
Potassium (mEq/L)	4.2	3.6-4.8
Chloride (mEq/L)	102	101-108
Total cholesterol (mg/dL)	150	142-248
HDL-cholesterol (mg/dL)	34	38-90
LDL-cholesterol (mg/dL)	104	65-163
C-reactive protein (U/L)	9.21	0.00-0.14
Blood sugar (mg/dL)	124	73-109
HbA1c (NGSP) (%)	6.4	4.9-6.0
Activated partial thromboplastin time (sec)	36.0	26.0-38.0
Prothrombin time (% in the normal range)	57.9	80.0-120.0
Fibrinogen (mg/dL)	>800	200-400

Although stent graft placement or artificial vessel replacement was considered, we opted for an extended course of antibiotic therapy for a minimum of two weeks, potentially up to a month, pending multiple negative blood cultures and normalization of inflammatory markers. Initially, antibiotics were administered to manage the infection, and a negative blood culture was achieved at our hospital, with subsequent blood tests showing significant improvement (Table 2).

**Table 2:** Preoperative laboratory analysis

Laboratory analysis	Results	Normal range
White blood cell counts (/μL)	6,500	3,300-8,600
Hemoglobin (g/dL)	12.4	13.7-16.8
Platelet count (×10^3^/μL)	221	158-348
Creatine kinase (U/L)	33	59-248
Lactate dehydrogenase (U/L)	202	124-222
Aspartate aminotransferase (U/L)	43	13-30
Alanine aminotransferase (U/L)	27	10-42
γ-glutamyl transpeptidase	25	13-64
Total bilirubin (mg/dL)	0.5	0.4-1.5
Direct bilirubin (mg/dL)	0.1	0.1-0.3
Alkaline phosphatase (U/L)	109	38-113
Amylase (mg/dL)	62	44-132
Creatinine (mg/dL)	0.83	0.65-1.07
Blood urea nitrogen (mg/dL)	21.9	8.0-20.0
Uric acid (mg/dL)	3.5	3.7-7.8
Sodium (mEq/L)	146	138-145
Potassium (mEq/L)	4.5	3.6-4.8
Chloride (mEq/L)	107	101-108
C-reactive protein (U/L)	0.22	0.00-0.14
Blood sugar (mg/dL)	96	73-109
Activated partial thromboplastin time (sec)	29.8	26.0-38.0
Prothrombin time (% in the normal range)	74.7	80.0-120.0
Fibrinogen (mg/dL)	387	200-400

**Figure 2:**
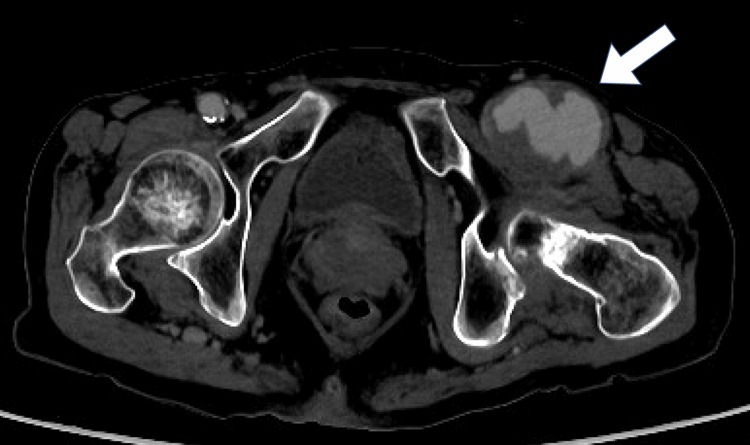
Enhanced CT at the time of emergency surgery reveals an enlarged pseudoaneurysm The allow indicates a pseudoaneurysm enlarged to 46 x 56 mm. CT: computed tomography

However, the aneurysm demonstrated clear signs of enlargement, necessitating emergency surgical intervention (Figure 2).

**Figure 3:**
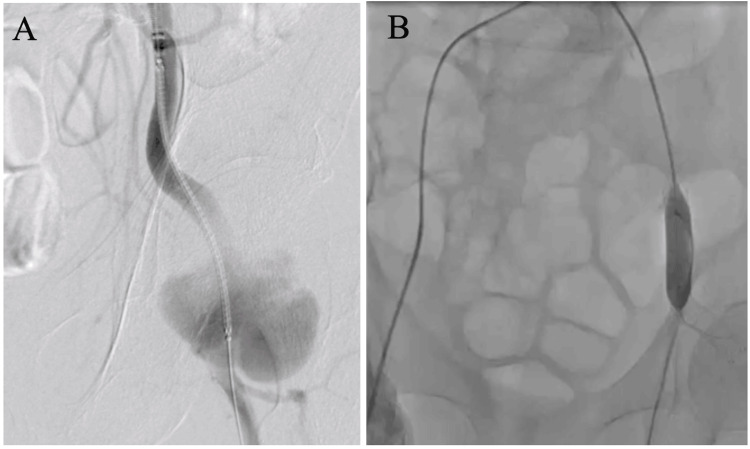
Hybrid repair performed in two stages A: Viabahn^®️ ^(10 x 100 mm) placed from the left external iliac artery to the common iliac artery. B: Mustang^®️ ^(10 x 40 mm) was used to block blood flow at the stent graft site and anastomosed with PROPATEN^®️ ^(8 mm).

Given the rapid expansion of the aneurysm and the associated risk of anastomotic failure due to the extensive replacement required, we selected a hybrid approach involving stent grafting from the external iliac artery to the proximal femoral artery, followed by artificial vessel replacement (Figure 3).

**Figure 4:**
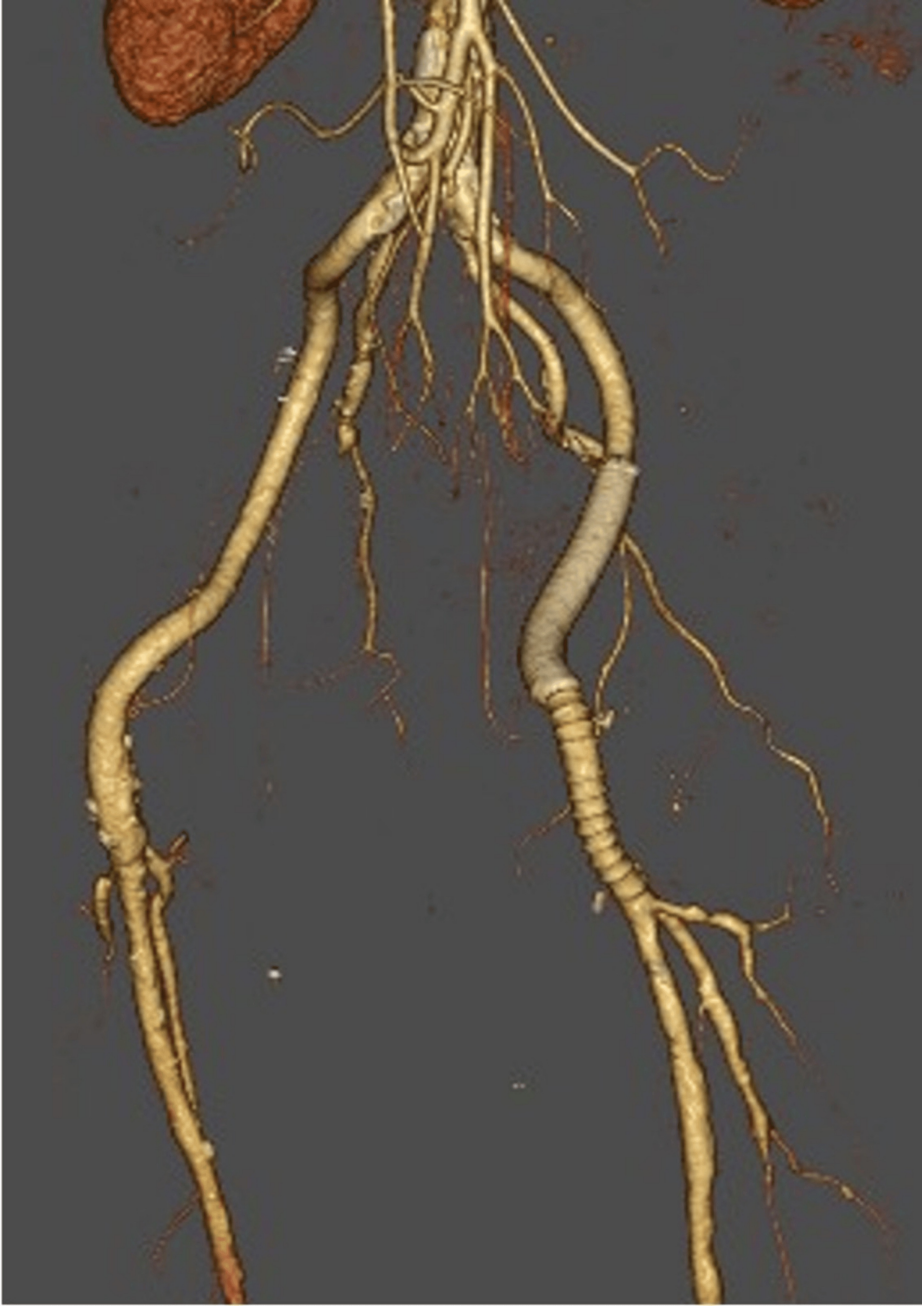
3D CT imaging demonstrates that the stent graft and artificial vessels remain intact and do not exhibit any signs of failure 3D: three-dimensional, CT: computed tomography

The infected pseudoaneurysm extended from the left external iliac artery to the CFA. Typically, such a procedure would require abdominal access to replace the external iliac artery, which is considerably more invasive. Instead, we employed a hybrid strategy that involved implanting a stent graft from the external iliac artery to the proximal CFA, followed by artificial vessel replacement from this point to the femoral artery bifurcation. The artificial vessel was end-to-end anastomosed on both sides. The postoperative course was uneventful, and follow-up enhanced CT imaging confirmed the absence of complications such as vascular collapse (Figure 4).

Twelve days post-surgery, the patient was transferred back to the previous hospital for continued rehabilitation due to discharge difficulties. Over a follow-up period of two months, the patient was discharged and appeared to be in good health. Unfortunately, the cancer had metastasized to his lungs and was progressing. Should the patient's condition worsen, palliative care will be considered. In this case, we successfully managed the infected pseudoaneurysm despite the impossibility of achieving a radical cure for cancer, demonstrating that hybrid therapy can be an effective strategy for patients who are not candidates for more invasive treatments.

## Discussion

The complication rate associated with IFAP is elevated, and the procedure is inherently highly invasive [2-4]. Endovascular stent-graft placement is an efficacious and minimally invasive alternative technique, particularly as a provisional measure for critically ill patients [5]. In this case, the patient was able to circumvent laparotomy and mitigate procedural invasiveness through a combination of artificial vessel replacement and stent-grafting techniques.

In previous reports, IFAPs are most frequently attributed to drug abuse. The standard procedure for IFAPs involves ligation of the CFA without subsequent revascularization, which, although common, poses significant risks of claudication, ischemia, and potential amputations [6]. Since drug-induced pseudoaneurysms are uncommon in our country, catheter procedures and femoral artery punctures are the primary causes. Given the high risk of ischemia and lower extremity amputation when collateral blood vessels are not well-developed, simple ligation of the CFA is not considered standard treatment. Treatment that includes revascularization is generally regarded as the standard of care. The principles of standard management dictate that the deployment of a covered stent is unlikely to succeed as a treatment option for infected pseudoaneurysms. This is because exclusion, rather than excision, of the pathology may result in a confined infection and contamination of the newly placed device, potentially leading to limb- and life-threatening consequences [7]. Emergency percutaneous stent graft implantation followed by secondary surgical repair was performed in this case. The stent graft was extended to the external iliac artery, with the peripheral side anastomosed to the artificial vessel.

If the anastomosis becomes infected, an anastomotic pseudoaneurysm could develop, posing a risk of life-threatening rupture. Stent grafting has been reported to be effective in treating infected anastomotic pseudoaneurysms [8]. There are also objections to placing stent grafts in all the common femoral arteries, and placement in designated "no stent zones" is not appropriate [9]. Consequently, we minimized the use of stent grafts in these areas by utilizing an artificial blood vessel.

## Conclusions

Although surgery remains the preferred procedure for repairing IFAPs, the combination of stent graft placement followed by pseudoaneurysm resection, surgical debridement, partial vascular replacement, and antimicrobial therapy has proven to be a viable, less invasive option in emergency pseudoaneurysm surgery. The results of this study are presented in the following sections: Hybrid surgery may be the most favored treatment for patients who are not suitable candidates for major surgery, in terms of both safety and efficacy.
